# High-fluoride toothpaste: a multicenter randomized controlled trial in adults

**DOI:** 10.1111/cdoe.12090

**Published:** 2014-12-20

**Authors:** Murali Srinivasan, Martin Schimmel, Martine Riesen, Alexander Ilgner, Michael J Wicht, Michael Warncke, Roger P Ellwood, Ina Nitschke, Frauke Müller, Michael J Noack

**Affiliations:** 1 Division of Gerodontology and Removable Prosthodontics, School of Dental Medicine, University of GenevaGeneva, Switzerland; 2 Department of Prosthodontics and Materials Science, Center for Dental, Oral and Maxillofacial Surgery, University of LeipzigLeipzig, Germany; 3 Department of Operative Dentistry and Periodontology, Centre of Dental Medicine, University of CologneCologne, Germany; 4 Medical Research Department, Colgate Palmolive CompanyHamburg, Germany; 5 Colgate Palmolive Dental Health Unit, Williams House, Manchester Science ParkManchester, UK; 6 Department of Rehabilitation and Geriatrics, University Hospitals of GenevaGeneva, Switzerland

**Keywords:** adult, dental caries, Duraphat 5000 ppm F, high-fluoride toothpaste, oral health, prevention, randomized controlled trial, root caries, sodium fluoride

## Abstract

**Objective:**

The aim of this single – blind, multicenter, parallel, randomized controlled trial was to evaluate the effectiveness of the application of a high-fluoride toothpaste on root caries in adults.

**Methods:**

Adult patients (*n* = 130, ♂ = 74, ♀ = 56; mean age ± SD: 56.9 ± 12.9) from three participating centers, diagnosed with root caries, were randomly allocated into two groups: Test (*n* = 64, ♂ = 37, ♀ = 27; lesions = 144; mean age: 59.0 ± 12.1; intervention: high-fluoride toothpaste with 5000 ppm F), and Control (*n* = 66, ♂ = 37, ♀ = 29; lesions = 160; mean age: 54.8 ± 13.5; intervention: regular-fluoride toothpaste with 1350 ppm F) groups. Clinical examinations and surface hardness scoring of the carious lesions were performed for each subject at specified time intervals (*T*_0_ – at baseline before intervention, *T*_1_ – at 3 months and *T*_2_ – at 6 months after intervention). Mean surface hardness scores (HS) were calculated for each patient. Statistical analyses comprised of two-way analysis of variance and post hoc comparisons using the Bonferroni–Dunn correction.

**Results:**

At *T*_0_, there was no statistical difference between the two groups with regard to gender (*P *=* *0.0682, unpaired *t*-test), or age (*P *=* *0.9786, chi-squared test), and for the overall HS (Test group: HS = 3.4 ± 0.61; Control group: HS = 3.4 ± 0.66; *P *=* *0.8757, unpaired *t*-test). The anova revealed significantly better HS for the test group than for the control groups (*T*_1_: Test group: HS = 2.9 ± 0.67; Control group: HS = 3.1 ± 0.75; *T*_2_: Test group: HS = 2.4 ± 0.81; Control group: HS = 2.8 ± 0.79; *P* < 0.0001). However, the interaction term time-point*group was not significant.

**Conclusions:**

The application of a high-fluoride containing dentifrice (5000 ppm F) in adults, twice daily, significantly improves the surface hardness of otherwise untreated root caries lesions when compared with the use of regular fluoride containing (1350 ppm F) toothpastes.

The advantageous effects of using low concentrations of fluorides (F) on enamel and dentin demineralization are very well documented 1. Fluoridated dentifrices, varnishes, and topical applications have contributed to a widespread decline in dental caries in most developed nations 2–4. Prophylactic fluoride applications have predominantly been indicated in children, and because of the increased risk of fluorosis, the fluoride content is restricted to a lower concentration 5–8. However, the use of low concentration fluoride may be ineffective in decreasing the caries susceptibility in high-risk subjects. High-fluoride interventions have been usually limited to chair-side topical professional applications. The use of high-fluoride containing varnishes or gels demonstrated a 40% improvement over stand-alone routine oral hygiene measures 9. Although, fluoride varnishes have demonstrated sufficient clinical evidence in the prevention and inhibition of dental caries, a Cochrane review reported that fissure sealants were more effective than varnishes and further emphasized that fluoridated toothpastes produce similar effects as with varnishes, gels, and mouth rinses 10.

Extensive clinical trials on fluoridated toothpastes have concluded that the fluoride content is an important factor in its effectiveness 3. Clinically, increased fluoride content results in increased fluoride levels in plaque films 11. It has been suggested that an increase of 500 ppm F within the range of 1100–2500 ppm F, results in an additional 6% caries reduction 12,13. Fluoride toothpastes with <1450 ppm F content have been reported to be less effective in high-risk children 14. Hence, it is highly likely that higher fluoride levels in toothpastes would decrease caries incidence more effectively. Adults with multiple coronal carious lesions or multiple root fillings are at increased risks of prevalent untreated root caries, especially elderly individuals with a compromised health status 15. Very few studies have reported the incidence of root caries in compromised adults 15–17. The prevalence of root caries is incidentally higher in elderly adults living in community-based residences or those diagnosed with dementia 17.

Most studies evaluating the effectiveness of fluoridated toothpastes have been conducted either in children or adolescents. The beneficial effect of high-fluoride toothpaste on dentin has already been demonstrated *in vitro*, preventing both mineral loss and lesion depth 18. A randomized control trial including adult dental school patients recruited with at least one root caries lesions has shown that high-fluoride toothpaste with 5000 ppm F was superior to a regular 1100-ppm F tooth gel 19. While another study has demonstrated that even regular toothpaste containing 1450 ppm F was beneficial in arresting root caries lesions 20. The elderly patients in this study however suffered from limited saliva function, although saliva secretion level had only poor predictive values for the preventive effect of the outcome. So there is still limited evidence about the superior effect of high-fluoride concentrations on arresting existing initial root caries lesions in an adult population.

The purpose of this clinical trial was to test the effectiveness of high-fluoride toothpaste (5000 ppm F) on root caries lesions in adults and to test the hypothesis that high concentration fluoride toothpaste would effectively improve the surface hardness in root caries lesions in adult patients.

## Methods

### Trial design

This single – blind, multicenter, parallel, randomized controlled trial with an allocation ratio of 1:1 was approved by the ethics committee of the University of Cologne in Germany (Approval No. 04 – 113), and notified with the BfArM (German Drug Regulatory Agency, Bonn, Germany). No modifications to the trial methods were performed after trial commencement. This randomized controlled trial is reported in accordance with the CONSORT (Consolidated Statement Of Reporting Trials) statement 21.

### Participants

Adult patients, diagnosed with untreated root caries, were to be included in the trial. The eligibility criteria for the recruitment of the study cohort are listed in Table1. This trial was conducted under university settings, and participants were recruited from the patient clinics of three dental schools located in Cologne (Germany), Leipzig (Germany) and Geneva (Switzerland).

**Table 1:** Predefined inclusion and exclusion criteria for the recruitment of subjects

Inclusion criteria
Age group between 18–75 years
Must have 10 or more natural teeth
Must have at least one root caries lesion
Teeth included in the study must not be crowned or compromised
Not having participated in another clinical trial in 6 months prior to this trial
Exclusion criteria
Medically unfit, or the presence of any hard or soft tissue tumors in the oral cavity
Patients undergoing radiation therapy
Ongoing fixed orthodontic appliance therapy
Open or active coronal decay
Undergone high-fluoride therapy 6 months prior to this trial
Local or systemic antibiotic therapy within the 6 months
Pregnancy, lactating, or hypersensitive to the trial test products
Acute progressive periodontitis

### Interventions

The patients were distributed in two groups (test and control groups). The patients in the test group were administered with toothpastes containing high-fluoride of 1.1% sodium fluoride (Duraphat 5000 ppm F; Colgate–Palmolive Company, Hamburg, Germany) in a silica substrate. The control group participants were allotted with standard regular-fluoride toothpaste (Odol-med 3, 1350 ppm F; GlaxoSmithKline, Brühl, Germany). Interventions were dispensed in separate packets to each patient according to their allotted groups. The packets were identical and consisted of three or four tubes of toothpastes along with two standard soft bristled adult toothbrushes.

### Outcome measures

The primary outcome measure determining the effectiveness of the test toothpaste was to evaluate the changes in the surface structure of the root caries lesions after the intervention. The following linear clinical surface texture grading scale was adopted for the evaluation of root caries surface hardness as validated in a former study 22:

- Level 1: Hard
- Level 2: Hard to Leathery
- Level 3: Leathery
- Level 4: Leathery with local softening
- Level 5: Soft

The surface hardness scores (HS) were recorded at:

- Baseline (*T*_0_)
- 3 months after intervention (*T*_1_), and
- 6 months after intervention (*T*_2_).

There were no changes to the outcome measures after trial commencement.

### Sample size

The sample size calculation was based on the findings from similar studies published in the literature 19,23. The desired statistical power was set at 80% (1 – β = 0.8) at a significance level of 5% (α < 0.05). Ninety subjects per group were required to avoid any statistical type II errors.

### Randomization and blinding

The random allocation sequence was generated manually in the Medical Research Department at Colgate–Palmolive Company in Hamburg, Germany. A block randomization was carried out (block size = 10; allocation ratio = 1:1). The randomly allocated sequence was implemented and concealed in sequentially numbered, consecutive, nontransparent sealed envelopes. The envelopes were maintained in the possession of each center's chief investigator. The envelopes were opened only prior to intervention after patient enrollment and after receiving the patient's consent. A single investigator from the medical research department at Colgate, who was not involved in the participants' enrollment process, generated the random allocation sequence. Consecutive patients who fulfilled the inclusion criteria were enrolled in the trial in each of the three centers. The examiners recruited the participants and were blinded to the participants' group allotments.

### Statistical methods

Statistical analyses were performed on a subject level and not on a surface level 24,25. The mean HS was calculated for each subject by dividing the sum of individual surface scores by the number of surfaces scored. This mean HS was recorded for *T*_0_, *T*_1_ and *T*_2._ The test and the control groups were compared at baseline in regard to age (unpaired *t*-test), gender (chi-squared test), and initial HS-score (unpaired *t*-test). Homogeneity of variances between groups was verified using F-tests (0.079 < *P *<* *0.8757). Subsequently, a two-way analysis of variance (anova) was performed to compare the HS scores of the two groups; with ‘group’ and ‘time point’ as independent variable. The interaction term was calculated and a post hoc Bonferroni–Dunn comparison with correction for repeated measures was performed. The level of significance was set to *P *<* *0.05. For the statistical analysis, SPSS 16.0 for Windows (SPSS Inc., Chicago, IL., USA) and StatView 5.0 for Windows (SAS Institute Inc., Cary, NC, USA) were used.

### Study protocol

A training meeting was conducted in Cologne for all the examiners in order to calibrate them to the patient recruitment and examination procedures, clinical scoring of the root surfaces, and data extraction procedures. Interoperator and intra-operator reliability was assured during this training session, and the applied probing pressure was also standardized. The interexaminer agreement of the clinical root caries scale was determined during the calibration meeting in Cologne where 19 patients were examined by all investigators. The unweighted kappa value was 0.84. Consecutive patients in each center qualifying based on the inclusion and exclusion criteria were screened and included in the study. After the initial review and examination, the patients were given detailed written information about the trial and a signed written consent was obtained from each patient who participated in the study. Baseline clinical examinations, including a complete oral health check-up, caries status, and plaque index were performed for all participants (Loe an Silness) 26. The surface hardness for the root caries lesions was graded and recorded with a standardized dental probe. This probe was standardized by type, model, and brand (Pluradent 43124 lot 20774; Offenbach, Germany), to be used for this study in all the three centers. A complete full mouth oral prophylaxis was performed, and oral hygiene instructions were given prior to the start of the study. The patients were then instructed on the use of the test products. Instructions were given to replace the toothbrushes provided every 6 weeks and were replaced with the same type of soft brushes as provided at the start of the study. During the study period, the patients received the same intervention packets every 3 months. Unused products were to be returned to the respective investigator during the examination visits at 3 and 6 months.

During the trial period, the patients were given specific instructions on brushing, that is, twice daily for 2 minutes with the toothbrushes and pastes provided. The quantity of the toothpaste to be used for each brushing procedure was approximated to about 1 g. Patients were strictly instructed to refrain from using toothpastes or toothbrushes other than the ones provided to them. They were also restricted from using mouth rinses. In contrast, no restrictions were made on the use of dental floss, interdental aids or denture cleansers during the trial period. No changes were recommended in their habits of smoking or diets during the study period. Patients were asked questions on their oral hygiene habits and were requested to fill in a final questionnaire at the end of the trial.

## Results

**Fig 1:**
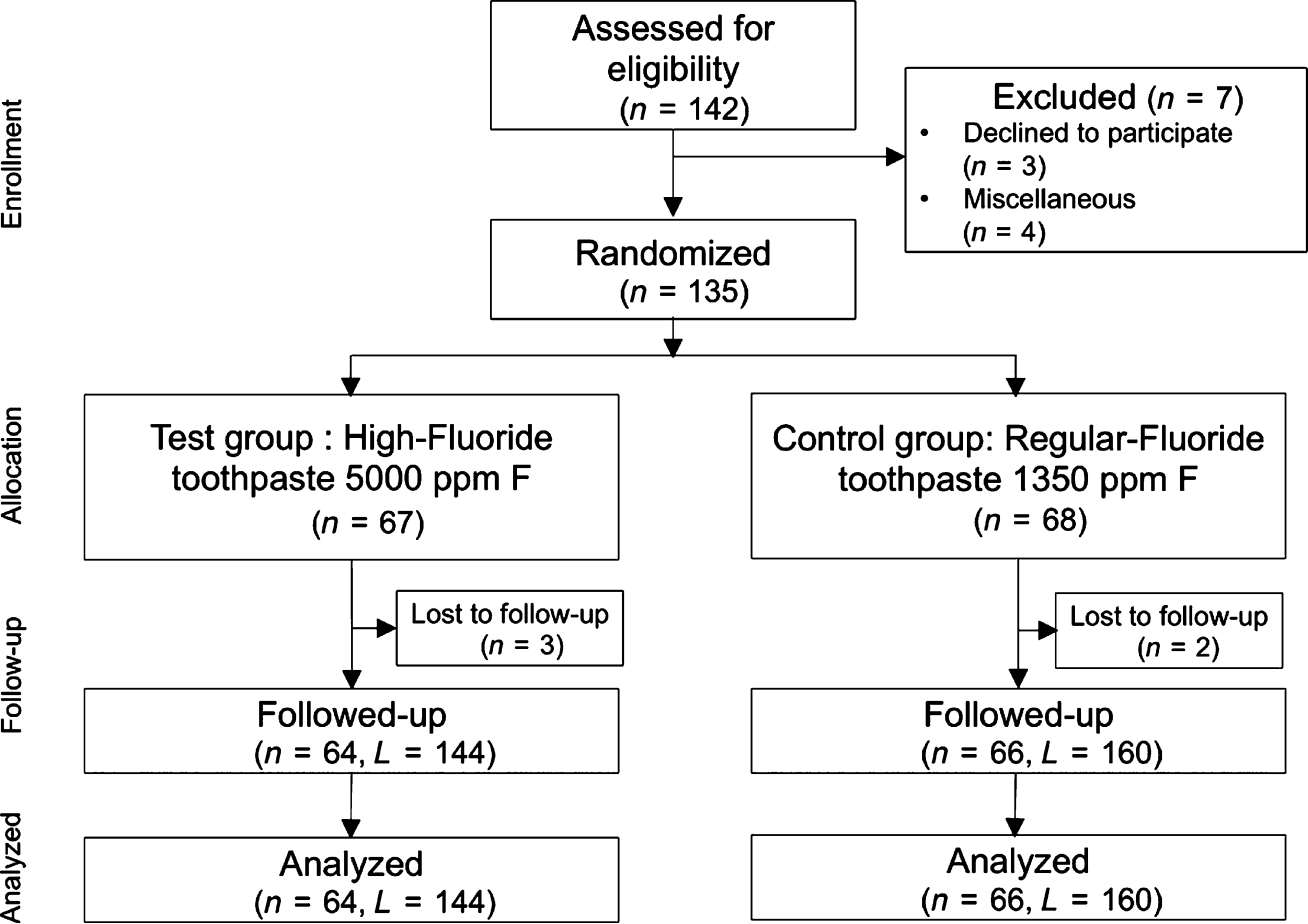
Flow chart of the phases of the two study groups in the trial (*n*, number of patients; L, number of root caries lesions).

A total of 142 patients were assessed for eligibility by the three participating centers. Seven patients were excluded and finally 135 patients (test group: *n* = 67; control group: *n* = 68) with 318 identified root caries lesions (L) were randomized and enrolled in the trial to receive the intended interventions. After trial commencement, five patients (test group: *n* = 3; control group: *n* = 2) were excluded from the study because they were lost to follow-up. Hence, a total of 130 patients (test group: *n* = 64, L = 144; control group: *n* = 66, L = 160) were included in the final analysis for the primary outcome measure. The *CONSORT* flow diagram of the phases in the participant recruitment, randomization, follow-up and analysis is presented (Fig.1). Recall examinations were performed at 3 (*T*_1_) and 6 months (*T*_2_) after intervention. The trial concluded after all the patients were examined at *T*_2,_ as originally intended in the original protocol.

The relevant patient demographics, group allocation and observations at *T*_0_ are presented (Table2). At *T*_0_, the two groups were not different with respect to age (*P *=* *0.0682, unpaired *t*-test), gender (*P *=* *0.9786, chi-squared test) and baseline mean HS values (*P *=* *0.8757, unpaired *t*-test).

**Table 2:** Baseline demographics of the study groups

Test center	Test group					Control group				
	Subjects (*n*)			Mean age (years)	Mean HS	Subjects (*n*)			Mean age (years)	Mean HS
	M	F	*T*			M	F	*T*		
Cologne	16	13	29	62.6 ± 8.3	3.5 ± 0.51	17	11	28	62.2 ± 8.6	3.5 ± 0.51
Leipzig	8	9	17	60.3 ± 10.9	3.5 ± 0.50	6	13	19	55.9 ± 10.7	3.5 ± 0.59
Geneva	13	5	18	51.8 ± 15.4	3.1 ± 0.75	14	5	19	43.1 ± 14.0	3.2 ± 0.89
Total	37**	27**	64	59.0 ± 12.1*	3.4 ± 0.61***	37**	29**	66	54.8 ± 13.5*	3.4 ± 0.66***

Test Group, High-fluoride toothpaste (5000 ppm F); Control Group, Regular-fluoride toothpaste (1350 ppm F); *n*, Number; M, Male; F, Female; *T*, Total; HS, Surface hardness score.

* *P *=* *0.0682 (unpaired *t*-test)

** *P *=* *0.9786 (chi-squared test)

*** *P *=* *0.8757 (unpaired *t*-test).

### Root caries surface hardness analysis

The changes in the HS for each of the individual study center are tabulated in Table3. The statistical model revealed a significant effect of the tested intervention (*P *=* *0.009, anova) and time (*P *<* *0.0001, anova) on the mean HS (Table4). However, the interaction term was not significant (group*time-point: *P *=* *0.1151, anova) as the HS improved within both the test and the control groups (*P *<* *0.0001, anova, *T*). The post hoc analysis (adjusted *P*-value for significance according to the Bonferroni–Dunn correction: *P *=* *0.0167) revealed that HS scores between the test and control groups were significantly different at the end of the observation period [*P *=* *0.0067(S), Bonferroni–Dunn], but not at *T*_0_ and *T*_1_ (*P *=* *0.8757 and *P *=* *0.1787, respectively, Bonferroni–Dunn). Furthermore, HS scores improved significantly in the test group between all time points (*T*_0_–*T*_1_: *P *=* *0.0008, *T*_1_–*T*_2_: *P *<* *0.0001, Bonferroni–Dunn). In the control group, the HS improved only after 3 months into the study (*T*_0_–*T*_1_: *P *=* *0.0358; *T*_1_– *T*_2_: *P *<* *0.0122; *P*-value: Bonferroni–Dunn).

**Table 3:** Changes observed in the mean surface hardness scores in the individual study centers

	Cologne			Leipzig			Geneva		
	Test group	Control group	Intergroup comparison^a^	Test group	Control group	Intergroup comparison^a^	Test group	Control group	Intergroup comparison^a^
Observation time									
T_0_	3.5 ± 0.51	3.5 ± 0.51	*P *= 0.8987	3.5 ± 0.50	3.5 ± 0.59	*P *= 0.8486	3.1 ± 0.75	3.2 ± 0.89	*P *= 0.7695
T_1_	2.8 ± 0.56	3.7 ± 0.60	*P *= 0.0764	3.3 ± 0.58	3.2 ± 0.69	*P *= 0.4447	2.8 ± 0.80	3.1 ± 1.02	*P *= 0.3211
T_2_	2.0 ± 0.57	2.7 ± 0.67	*P *< 0.0001	3.0 ± 0.57	2.7 ± 0.65	*P *= 0.2226	2.5 ± 0.91	3.0 ± 1.08	*P *= 0.1610
Intragroup comparison^a^									
T_0_ versus T_1_	*P *< 0.0001	*P *= 0.0089		*P *= 0.3693	*P *= 0.1484		*P *= 0.4212	*P *= 0.9921	
T_0_ versus T_2_	*P *< 0.0001	*P *< 0.0001		*P *= 0.0134	*P *= 0.0010		*P *= 0.0654	*P *= 0.6816	
T_1_ versus T_2_	*P *< 0.0001	*P *= 0.0161		*P *= 0.1031	*P *= 0.0492		*P *= 0.2886	*P *= 0.6889	

Test Group, High-fluoride toothpaste (5000 ppm F); Control Group, Regular-fluoride toothpaste (1350 ppm F); *T*_0_, Baseline; *T*_1_, 3 months; *T*_2_, 6 months.

a *Post hoc* Bonferroni–Dunn correction for multiple comparisons, adjusted *P*-value for significance *P *=* *0.0167.

**Table 4:** Changes observed in the combined mean surface hardness scores (all centers)

	Test group	Control group	Intergroup comparison^a^
Observation time			
* T*_0_	3.4 ± 0.61	3.4 ± 0.66	*P *=* *0.8757
* T*_1_	2.9 ± 0.67	3.1 ± 0.75	*P *=* *0.1787
* T*_2_	2.4 ± 0.81	2.8 ± 0.79	*P *=* *0.0067
Intragroup comparison^a^			
* T*_0_ versus *T*_1_	*P *=* *0.0008	*P *=* *0.0358	
* T*_0_ versus *T*_2_	*P *<* *0.0001	*P *<* *0.0001	
* T*_1_ versus *T*_2_	*P *<* *0.0001	*P *=* *0.0122	

Test Group, High-fluoride toothpaste (5000 ppm F); Control Group, Regular-fluoride toothpaste (1350 ppm F); *T*_0_, Baseline; *T*_1_, 3 months; *T*_2_, 6 months.

Significant treatment effect (*P *=* *0.0090, anova) and effect of time-point (*P *<* *0.0001); the interaction term treatment^*^time not significant (*P *=* *0.1151).

a Post hoc Bonferroni–Dunn correction for multiple comparisons, adjusted *P*-value for significance *P *=* *0.0167.

## Discussion

Although the methodology followed in this study protocol is robust, a few limitations do exist. Due to technical reasons, it was not possible to produce identical packages for the test and control toothpaste. Consequently, the patients knew whether they were in the test or in the control group even though they were supposed to be blinded to the interventions. However, the examiner was blinded to the patient's group assignment. Another shortcoming was the failure to recruit the sample size proposed by the power calculation 27. The power was not adequate to demonstrate that the high-fluoride group improved surface hardness better than the low-fluoride group over time. This may have been better demonstrated with a larger sample size. However, a surface level analysis could have been performed in this study to increase the power of the study. This was not performed because surface level data cannot be considered as independent observations. Nevertheless, the sample size of the current study was sufficient to demonstrate a significant difference between the interventions groups without taking the time point into consideration. The duration of this clinical study was limited by expiry date of the test product, and these products were all provided immediately after ethical approval. Hence, the recruitment process was thus shortened and consequently, smaller than intended sample size. Despite a carefully planned calibration meeting, differences might have occurred between the study centers in performing the protocol or in the features of the enrolled patients and therefore in the results obtained. However, these differences did not preclude the significant differences between the test and control groups after an observation period of 6 months. Due to our stringent patient selection criteria resulting in the exclusion of compromised individuals, the generalizability of the results may be limited.

Numerous studies are present in current literature, which highlight the benefits of using fluoridated dentifrices in the prevention and treatment for dental caries in children and young adults 11,28–30. Very few studies have focused on the potential benefits of using fluoridated toothpastes in adults, more specifically the compromised elderly adults, and even fewer studies have validated the efficacy of fluoridated dentifrices on untreated adult root caries 15–17. Former works have published that elderly population who are medically compromised have increased plaque levels compared with normal healthy adults 16,31,32. Consequently, there is a higher potential for the incidence of caries in such adults 33–36. Moreover, currently recommended standard fluoridated toothpastes show a fluoride content of 1100–2500 ppm F 12–14. Increased fluoride content in dentifrices has been proven to reduce the incidence of caries 12,13.

The beneficial effect of a high-fluoride content (5000 ppm F) dentifrice has already been studied *in vitro* on bovine enamel 18,37 and on root caries lesions in some clinical studies evaluating patients with compromised periodontal health 38 and elderly patients 19,20,23,39,40. The results of our study confirm the results of earlier studies for an adult population with root caries risk. Although the reported differences are statistically significant, yet the SD indicates a large variability of the obtained improvements between participants. The clinical relevance may therefore be more important for some participants than for others. Our study highlights the preventive effect of the high-fluoride toothpaste, but further trials are necessary to confirm this effect.

The rationale of using a dentifrice with a high-fluoride concentration is of particular importance in the elderly population, where oral hygiene measures are difficult because of impeded vision, tactile sensitivity, as well as a reduced dexterity 16. In addition, multimorbid patients often present little motivation to perform extended oral hygiene measures, when their general health status is compromised and disease and/or functional impairment dominate their daily life. It has also to be considered that the restorative measures are less often accepted by these patients due to not only a lack of motivation, but also of financial resources. From a clinician's perspective, these restorative measures are often compromised or impossible, when the lesions extend into the proximal and sub gingival surfaces of the root. In a geriatric context, rubber dam is often difficult to apply, which compromises adhesive techniques. Even where mechanical undercuts can be achieved, the application of filling material is challenging when the lesion extends to the proximal root surfaces or even the lingual aspects of the tooth. Thus, there is a clinical need in nonrestorative treatment concepts for these carious root lesions. Ozone application has been suggested for this very purpose, but it requires a major financial investment and additional chair-side time 41–43. The great disadvantage of this therapy is that it cannot be used as a daily measure by the patient himself or the caretaker and depends on access to professional dental services. With the high-fluoride toothpaste, it is easy to administer and does not require elaborate equipment, investment, or professional support. The patients are usually familiar with the use of toothpaste, and such familiar circumstances preclude psychological apprehension toward its application. However, it has to be borne in mind that the institutionalized population presents a high prevalence of dysphagia and other swallowing disorders 44. Due to the aspiration risk, in patients with dysphagia and in ventilated patients, the use of fluoridated toothpastes may not be the first line of prevention for oral infection and caries. A final shortcoming of the proposed treatment for root caries is that it takes up to 6 months to produce the desired results. During this period of time, the required discipline and consistency to achieve clinical hardening of the root surface lesions may exceed the effort an elderly person is ready to invest. The results of this study do however suggest that the surface hardness of the root caries lesions improved in both groups (control and test), that is, both toothpastes improved the mean hardness scores of the lesions, in a time frame of 6 month usage.

## Conclusions

The application of a high-fluoride containing dentifrice (5000 ppm F) in adults, twice daily, significantly improves the surface hardness of untreated root caries lesions when compared with the use of regular fluoride containing (1350 ppm F) toothpastes. The potential application of such a product is particularly beneficial in improving oral health and reducing root caries susceptibility in elderly adults.
